# Serum proteomic-based analysis identifying autoantibodies against PRDX2 and PRDX3 as potential diagnostic biomarkers in nasopharyngeal carcinoma

**DOI:** 10.1186/s12014-017-9141-5

**Published:** 2017-02-01

**Authors:** Lie-Hao Lin, Yi-Wei Xu, Li-Sheng Huang, Chao-Qun Hong, Tian-Tian Zhai, Lian-Di Liao, Wen-Jie Lin, Li-Yan Xu, Kai Zhang, En-Min Li, Yu-Hui Peng

**Affiliations:** 1 Department of Clinical Laboratory Medicine, The Cancer Hospital of Shantou University Medical College, No. 7, Raoping Road, Shantou, 515041 Guangdong China; 2 Department of Orthopaedics, The Nanao People’s Hospital, Shantou, 515999 China; 3 The Key Laboratory of Molecular Biology for High Cancer Incidence Coastal Chaoshan Area, Shantou University Medical College, Shantou, 515041 China; 4 Department of Radiation Oncology, The Cancer Hospital of Shantou University Medical College, Shantou, 515041 China; 5 Department of Oncological Laboratory Research, The Cancer Hospital of Shantou University Medical College, Shantou, 515041 China; 6 Institute of Oncological Pathology, Shantou University Medical College, Shantou, 515041 China; 7 Tianjin Key Laboratory of Medical Epigenetics, Department of Biochemistry and Molecular Biology, School of Basic Medical Sciences, Tianjin Medical University, Tianjin, 300070 China; 8 Department of Biochemistry and Molecular Biology, Shantou University Medical College, Shantou, 515041 China

**Keywords:** Serological proteome analysis, Autoantibody, Peroxiredoxin, Early diagnosis, Nasopharyngeal carcinoma

## Abstract

**Background:**

Nasopharyngeal carcinoma (NPC) is a major head and neck cancer with high occurrence in Southeast Asia and southern China. We aimed to identify autoantibodies that may contribute to early detection of NPC.

**Methods:**

We used serological proteome analysis to identify candidate autoantibodies against tumor-associated antigens. Levels of autoantibodies and Epstein–Barr virus capsid antigen-IgA (VCA-IgA) were measured by ELISA in 129 patients with NPC and 100 normal controls. We employed receiver operating characteristics to calculate diagnostic accuracy.

**Results:**

Sera from patients with NPC yielded multiple spots, two of which were identified as PRDX2 and PRDX3. Levels of serum autoantibodies against PRDX2 and PRDX3 were significantly higher for patients with NPC than for normal controls (*P* < 0.01), respectively. Combined detection of autoantibodies against PRDX2 and PRDX3 and VCA-IgA provided a high diagnostic accuracy in NPC (an area under the curve (AUC) of 0.811 (95% CI 0.753–0.869), 66.7% sensitivity, and 95.0% specificity). This combination maintained diagnostic performance for early NPC with AUC value of 0.754 (95% CI 0.652–0.857), 50.0% sensitivity, and 95.0% specificity.

**Conclusions:**

This study reports autoantibodies against PRDX2 and PRDX3 identified by a proteomic approach in sera from NPC patients. Our findings suggest that autoantibodies against PRDX2 and PRDX3 may serve as supplementary biomarkers to VCA-IgA for the screening and diagnosis of NPC.

## Background

Nasopharyngeal carcinoma (NPC) is one of the most common tumors in the head and neck, with 86,500 new cases in 2012 worldwide [1]. The geographical distribution of NPC is very unique, with 71% of all new cases in east, southeast Asia and the remainder in south-central Asia, and north and east Africa [1]. Besides difference in geographical condition, some ethnic groups including the Nagas in northern India, the Bidayuh in Borneo, and Inuits in the Artic, also appear to have a predisposition for NPC [2]. In the aspect of the demographics, men are two to three times more likely to suffer from NPC than are women, and peak incidence is between the ages of 50 and 60 years [1]. The overall survival in early-stage NPC patients is obviously longer than that in patients with advanced stage [3–5]. The 5 years’ survival rate for stage IVA, B and C patients were only 67, 68 and 18%, respectively, while it could reach up to 100 and 95% for stage I and II patients after treatment, respectively [5]. Moreover, treatment-related morbidities more frequently occur in those patients with advanced disease [3]. However, due to deep anatomical site and the lack of specific symptoms at early stage, 75–90% of patients with NPC present with late stage of disease at clinical diagnosis [3, 6]. Thus, early diagnosis based on biomarker screening method may contribute significantly to NPC therapy and prognosis.

NPC is closely associated with Epstein–Barr virus (EBV), which is present in almost every NPC case, regardless of geographic distribution and histologic differentiation [7–9]. At present, the attempts for early NPC diagnosis mainly depends on various EBV-derived/related factors. EBV viral capsid antigen immunoglobulin A (VCA-IgA) and EBV DNA, the most commonly used serum/plasma biomarkers for NPC, have been found to be not sensitive and specific enough for early diagnosis purpose [10–12]. A recent meta-analysis showed that the sensitivity and specificity of VCA-IgA in diagnosis of NPC were 83 and 85%, respectively, and they were 75 and 87% for EBV DNA [12]. Other effective biomarkers for improving early NPC detection are thus clearly needed. In recent years, autoantibodies against tumor-associated antigens (TAAs) as serum biomarkers show potential availability for early cancer diagnosis, which could be detected prior to the onset of cancer [13–19]. Our previous studies also indicated that autoantibodies could serve as possible biomarkers for early detection of NPC [13, 14]. Here, we used a proteomic-based approach and identified novel TAAs PRDX2 and PRDX3 that induces an antibody response in patients with NPC.

## Methods

### Study design and participants

Approval for the study from the institutional ethics review committee center was obtained, and written informed consents were obtained from all patients and normal controls.

We included 7 NPC samples and 7 control samples in the “discovery” stage of this study (i.e. the serological proteome analysis, SERPA), who were consecutively collected from the Cancer Hospital of Shantou University Medical College, China, in February 2015. To further validate our findings in the SERPA, we performed the enzyme-linked immunosorbent assay (ELISA) in the validation stage using 129 NPC patients and 100 normal controls, which were recruited consecutively in the Cancer Hospital of SUMC from July, 2014, to July, 2015 and from the healthy staff members of this hospital between April, 2012, and June, 2014, respectively. The participants’ features are summarized in Table 1. NPC was defined and biopsy proven as described in our previous study [14]. Tumor stage was defined according to the seventh edition of the UICC/AJCC staging system for NPC [20].

**Table 1:** Basic patient demographics

	Discovery stage		Validation stage	
	NPC	Control	NPC	Control
Number	7	7	129	100
Gender				
Male	5	5	100	41
Female	2	2	29	59
Mean age ± SD (years)	59 ± 12	59 ± 10	51 ± 12	51 ± 10
Age range (years)	38–76	40–74	19–76	24–79
T stage				
T1	0		19	
T2	2		46	
T3	3		39	
T4	2		25	
N stage				
N0	1		17	
N1	1		50	
N2	5		55	
N3	0		7	
M stage				
M0	6		122	
M1	1		7	
Overall stage				
I	0		4	
II	0		36	
III	4		55	
IV	3		34	

The recruited patients were all newly diagnosed. We classified tumors with AJCC stage I + II as early-stage NPC as reported previously [13]. Details for blood sample collection, processing and storage of serum sample of all participants were described in our previous publication [13].

### Cell line

Human NPC cell line CNE2, obtained from Sun Yat-sen University Cancer Center, was cultured in RPMI-1640 medium plus 10% fetal bovine serum. All cells were incubated at 37 °C under an atmosphere of 5% CO_2_.

### Two-dimensional polyacrylamide gel electrophoresis and Western blotting

To discover novel autoantibodies in NPC patients, we followed the approach as described previously [21]. Protein extracts from cultured cells were diluted with two-dimensional gel electrophoresis (2-DE) sample buffer (8 M urea, 4.0% CHAPS, 0.2% (w/v) Ampholyte, 65 mM DTT), actively rehydrated into 11 cm, pH 3–10 nonlinear ReadyStrip™ IPG Strips (Bio-Rad, Hercules, California) by incubating for 15 h at 20 °C in a strip holder, and subjected to isoelectric focusing gel electrophoresis (the first-dimension gel). We performed the isoelectric focusing in the PROTEAN IEF cell at 250 V for 30 min, 1000 V for 30 min, 8000 V for 4 h, and 8000 V for 40,000 V-h. After focusing, the IPG strips were incubated for 15 min with equilibration solutions (375 mM Tris–HCl (pH 8.8) containing 6 M urea, 20% (w/v) glycerol, and 2.0% (w/v) SDS) supplemented with 10 mg/ml DTT and 25 mg/ml iodoacetamide. The treated gel strips were loaded onto the second-dimension gel, after which the gels were stained using a Pierce Silver Stain Kit (Thermo, Waltham, MA) or transferred onto a Hybond P polyvinylidene fluoride (PVDF) membrane using the iBlot^®^ 2 Dry Blotting System (Thermo). After transfer, PVDF membranes were blocked with blocking buffer (PBS/0.05% Tween 20 with 5.0% nonfat dry milk), and then incubated overnight at 4 °C with diluted sera from NPC patients or normal controls at 1:250 dilution. After washing, membranes were incubated with horseradish peroxidase (HRP)-conjugated goat anti-human IgG (Santa Cruz Biotechnology, Dallas, Texas) at 1:6000 dilution for 1.5 h at room temperature. Bound antibodies were detected by luminal reagent.

### In-gel digestion and purification of peptides

Bands were excised from the gels and subjected to in-gel tryptic digestion. Briefly, the silver staining bands were excised and washed with 10% acetic acid/50% ethanol for overnight, and further soaked in water for 20 min. The gel slices were cut into 1 × 1 mm pieces after destained with 100 mM potassium ferricyanide and 30 mM sodium thiosulfate. The gel slabs were washed with water, 25 mM ammonium bicarbonate in ethanol/water and acetonitrile, respectively. After completely dehydrated, it was dried using the SpeedVac. Each sample was further subjected to reduction and alkylation as following procedure. DTT was added into the sample (final concentration of 5 mM) and incubated at RT for 45 min. Iodoacetamide was added at a final concentration of 10 mM and incubated at RT for 30 min in the dark. After rehydrated, the gel particles were incubated with trypsin solution (10 μg/ml in 50 mM ammonium bicarbonate) at 37 °C overnight. The in-gel digests were extracted and concentrated to complete dryness using the SpeedVac and stored at −20 °C.

### Nano-HPLC–MS/MS analysis and data interpretation

The resulting samples were desalted using a C18 ZipTip (Millipore Corporation, Billerica, Massachusetts, USA), respectively, prior to Nano-LC–MS/MS analysis. Each tryptic digestion was reconstituted in 5 µL of LC buffer A (0.1% (v/v) formic acid in water and injected into a Nano-LC system (EASY-nLC 1000, Thermo Fisher Scientific, Waltham, MA). The peptides were separated by a C18 column (50 μm inner-diameter × 15 cm) with a 60 min HPLC-gradient (linear gradient from 2 to 35% HPLC buffer B (0.1% formic acid in acetonitrile) in 50 min, and then to 90% buffer B in 10 min). The HPLC elution was electrosprayed directly into a Q Exactive mass spectrometer (Thermo Fisher Scientific, Waltham, MA). The mass spectrometric analysis was carried out in a data-dependent mode, and the parameters were set as followings. The voltage at source was 1.8 kV. For full MS, scan range was from 350 to 1750 with the resolution of 70,000. The 10 most intense peaks with charge state 2 or 3 were selected for MS2 analysis (higher-energy collision dissociation: normalized collision energy of 27%, the resolution of MS2: 17,500 resolution, the dynamic exclusion duration for the data-dependant scan: 18 s, the repeat count: 2, and the exclusion window: ±1.5 Da). All MS/MS spectra were searched against the Uniprot-Human protein sequence database using the PD search engine (version 2.1.0, Thermo Fisher Scientific) with an overall false discovery rate (FDR) for peptides of less than 1%. Trypsin was specified as digesting enzyme. A maximum of 2 missing cleavages was allowed. Mass tolerances for precursor ions were set at ±10 ppm for precursor ions and ±0.02 Da for MS/MS. Oxidation of methionine and acetylation on protein N-terminal were fixed as variable modifications. Carbamidomethylation on Cys was specified as fixed modification. All MS/MS spectra were manually verified.

### ELISA for autoantibodies

ELISA for autoantibodies against PRDX2 and PRDX3 was performed by two researchers (Yi-Wei Xu and Lie-Hao Lin) as previously described [13, 18]. Briefly, purified recombinant antigens, PRDX2 (Sino Biological Inc.) and PRDX3 (Abcam, ab168006) were diluted to a final protein concentration of 0.1 and 0.3 μg/ml, respectively. 100 μl of serum samples and quality control samples (i.e. a pooled serum sample collected randomly from 50 patients with NPC) were diluted (1/110), added to the plates, as well as appropriate control rabbit polyclonal antibodies specific for capture proteins (rabbit anti-PRDX2 polyclonal antibody, Sino Biological Inc; rabbit anti-PRDX3 polyclonal antibody, Sino Biological Inc). Horseradish peroxidase (HRP)-conjugated goat anti-human/rabbit IgG (Santa Cruz Biotechnology) was added at 1:10,000 dilution.

Quality control for monitoring of the ELISA assay was conducted according to our previous study [13, 18].

### ELISA for EBV VCA-IgA

Concentrations of VCA-IgA in all samples were determined in duplicate by ELISA using commercial kits (Berer Bioengineering, Beijing, China). We conducted the experiments according to the manufacturer’s instructions as previously described [13, 14].

### Statistical analysis

We used the Mann–Whitney *U* test for analyses that compared levels of individual autoantibodies in serum between NPC patients and normal controls. For diagnostic ability of individual autoantibodies and jointly biomarker, we plotted receiver operating characteristic (ROC) analysis to assess optimum cutoff value, area under the ROC curve (AUC) with 95% confidence interval (CI), sensitivity, and specificity. The optimum cutoff value for positive reactivity was determined by achieving the maximum sensitivity when the specificity was >95%, and by minimizing the distance of the cutoff value to the top-left corner of the ROC curve. To test the diagnostic accuracy when the different markers were combined, we estimated functions of the combined markers by binary logistic regression, and the values of these functions were used as one marker and subjected to ROC analysis [22]. We used Chi-squared tests or Fisher’s exact tests for comparisons of the clinical relevance of individual and combined tests. All statistical analyses were performed with SPSS (version 17.0), or GraphPad Prism software. We considered a p value (two sided) of lower than 0.05 to be statistically significant.

## Results

### Identification of autoantibodies by serological proteome analysis

**Fig. 1:**
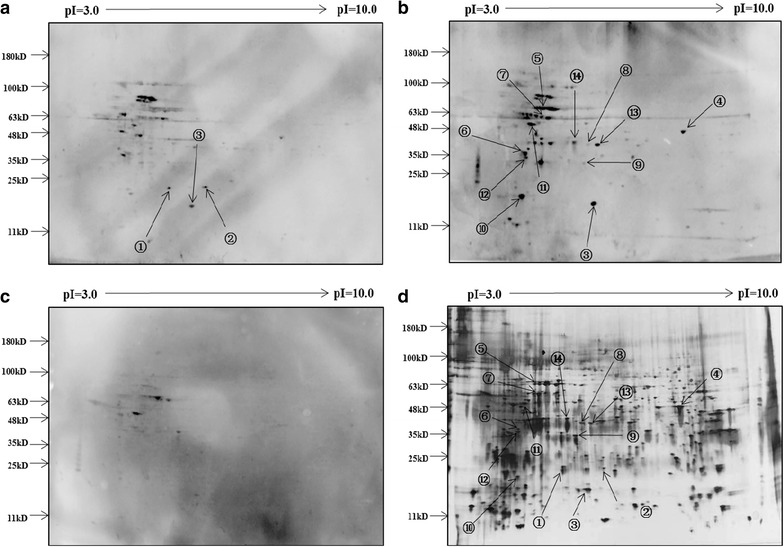
Representative two-dimensional protein gel of CNE2 cell lysate proteins with accompanying western blots. **a** CNE2 cell lysate proteins were separated by two-dimensional PAGE, transferred to PVDF membrane, and then incubated with diluted sera (1:250) from a patient with NPC. **b** PVDF membrane incubated with sera from another patient with NPC. **c** PVDF membrane incubated with sera from a normal control. PVDF membranes were incubated with appropriate secondary antibodies and visualized by chemiluminescence. **d** Silver stained two-dimensional gel for total protein isolated from the CNE2 NPC cell line

Human NPC CNE2 cell proteins were separated by 2-DE, and transferred onto PVDF membranes or visualized by silver staining (Fig. 1d). The membranes were screened individually from 7 NPC patients and from 7 matched normal controls to identify the presence of autoantibodies against candidate antigens from CNE2 cells. We selected 14 reactive spots in total observed in NPC patients for identification using the Nano-HPLC–MS/MS (Fig. 1a, b). Meanwhile there were no such reactive spots (or spots with weak immunoreactivity) within 7 healthy controls (Fig. 1c). We also observed that each selected target identified by the Nano-HPLC–MS/MS analysis correlated highly to the predicted molecular mass on gel from which it was originally excised (Table 2). Among these reactive spots, spot numbers 1 and 2, which were observed in 2 and 3 of 7 NPC patients, respectively (Fig. 1a), were identified as PRDX2 and PRDX3, respectively (Table 2). Both of the autoantibody biomarkers were selected to evaluate the diagnostic value for NPC with use of a validation cohort.

**Table 2:** List of tumor proteins detected by proteomic identification

Spot no.	Proteins	Accession no.	Molecular weight (kDa)	Mascot score	Sequence coverage (%)
1	PRDX2	P32119	21.9	45.25	27.27
2	PRDX3	P30048-2	25.8	61.26	26.05
3	Nucleoside diphosphate kinase A	P15531	17.1	319.20	50.66
4	ENO1	P06733	47.1	1101.89	63.36
5	HSPA8	P11142	70.9	166.27	31.42
6	Serine-threonine kinase receptor-associated protein	Q9Y3F4	38.4	149.54	32.86
7	HSPD1	P10809	61.0	724.87	38.57
8	Serpin B5	P36952	42.1	115.84	19.47
9	L-lactate dehydrogenase B	P07195	36.6	428.81	35.93
10	Gamma-glutamylcyclotransferase	O75223	21.0	40.35	20.74
11	Vimentin	P08670	53.6	177.94	45.06
12	NPM1	P06748-2	29.4	233.66	22.64
13	Isoform 2 of Macrophage-capping protein	P40121-2	36.8	47.09	10.21
14	Annexin A2	P07355	38.6	208.50	59.59

### Validation of autoantibodies against PRDX2 and PRDX3 for NPC

**Fig. 2:**
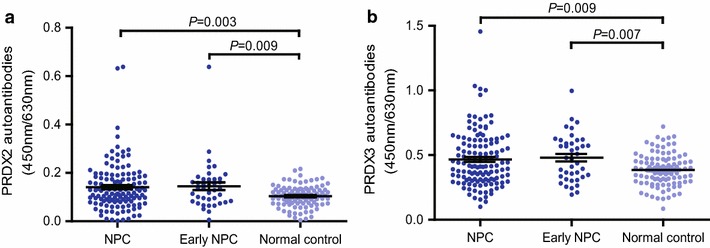
Levels of autoantibodies against PRDX2 and PRDX3. Scatter plots of OD values of autoantibodies against PRDX2 and PRDX3 from NPC sera and normal sera. *Black horizontal lines* are means

**Fig. 3:**
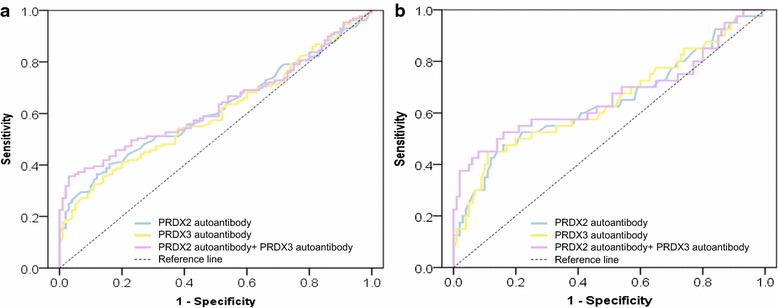
ROC curve analysis of autoantibodies against PRDX2 and PRDX3 for NPC diagnosis. **a** ROC curve for serum autoantibodies against PRDX2 and PRDX3 and their combination for patients with NPC versus normal controls. **b** ROC curve for serum autoantibodies against PRDX2 and PRDX3 and their combination for patients with early NPC versus normal controls. *ROC* receiver operating characteristic

In the validation stage, the circulating levels of autoantibodies against PRDX2 and PRDX3 on ELISA were significantly higher in NPC cases, respectively, compared with control individuals (Fig. 2, *P* < 0.01). ROC curve showed that the cutoff values of autoantibody against PRDX2 and PRDX3 were 0.173 and 0.608, respectively, with sensitivities/specificities of 26.4/96.0, 24.5/95.0%, and AUC values of 0.614 (95% CI 0.542–0.686) and 0.600 (95% CI 0.528–0.673) for discriminating NPC from normal controls (Fig. 3, Table 3). Predictive values and likelihood ratios for autoantibodies against PRDX2 and PRDX3 in the diagnosis of NPC are also shown in Table 3.

**Table 3:** Measurement of PRDX2 autoantibody, PRDX3 autoantibody and their combination of VCA-IgA in NPC diagnosis

	AUC (95% CI)	Sensitivity (%)	Specificity (%)	PPV (%)	NPV (%)	PLR	NLR
NPC versus NC							
PRDX2 autoantibody	0.614 (0.542–0.686)	26.4	96.0	89.5	50.3	6.60	0.77
PRDX3 autoantibody	0.600 (0.528–0.673)	24.5	95.0	86.3	49.4	4.90	0.79
PRDX2 autoantibody + PRDX3 autoantibody	0.632 (0.561–0.703)	36.4	95.0	90.4	53.7	7.28	0.67
VCA-IgA	0.719 (0.653–0.785)	48.8	95.0	92.6	59.0	9.76	0.54
Autoantibody + VCA-IgA	0.811 (0.753–0.869)	66.7	95.0	94.5	68.9	13.34	0.35
Early-stage NPC versus NC							
PRDX2 autoantibody	0.642 (0.532–0.753)	27.5	96.0	73.4	76.8	6.88	0.76
PRDX3 autoantibody	0.646 (0.537–0.755)	25.0	95.0	66.7	76.0	5.00	0.79
PRDX2 autoantibody + PRDX3 autoantibody	0.664 (0.550–0.779)	40.0	95.0	76.2	79.8	8.00	0.63
VCA-IgA	0.638 (0.528–0.747)	32.5	95.0	72.3	77.8	6.50	0.71
Autoantibody + VCA-IgA	0.754 (0.652–0.857)	50.0	95.0	80.0	82.6	10.00	0.53

*AUC* area under curve, *95% CI* 95% exact confidence interval, *NPC* nasopharyngeal carcinoma, *NC* normal controls, *PPV* positive predictive value, *NPV* negative predictive value, *PLR* positive likelihood ratio, *NLR* negative likelihood ratio

To estimate the diagnostic ability of the combined use of the two autoantibody markers, a variable predicted probability (*p*) of being detected as NPC was created based on an equation obtained by binary logistic regression (all NPC vs. all controls): ln (*p*/(1 − *p*)) = 6.040 × (PRDX2) + 2.062 × (PRDX3) − 1.339. The efficacy of combination of autoantibodies against PRDX2 and PRDX3 is presented in Fig. 3 and Table 3. Use of the combination of autoantibodies against PRDX2 and PRDX3 provided a sensitivity of 36.4% and a specificity of 95.0%.

### Combined detection of autoantibodies and VCA-IgA for NPC

**Fig. 4:**
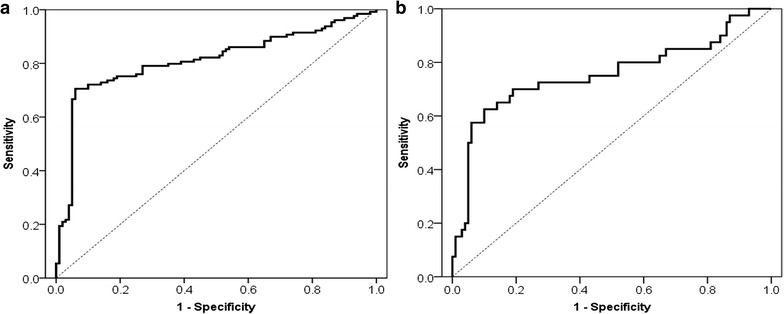
ROC curve analysis of the combination of autoantibodies against PRDX2 and PRDX3, and VCA-IgA. **a** ROC curve for the combination of autoantibodies against PRDX2 and PRDX3, and VCA-IgA for patients with NPC versus normal controls. **b** ROC curve for the combination of autoantibodies against PRDX2 and PRDX3, and VCA-IgA for patients with early NPC versus normal controls. *ROC* receiver operating characteristic; NPC

According to the manufacturer’s instructions, the recommended clinical cutoff value of VCA-IgA was 0.150. The sensitivities/specificities of VCA-IgA for all NPC and early-stage NPC were 48.8/95.0 and 32.5/95.0%, respectively (Table 3). We then examined whether the combined detection of VCA-IgA and autoantibody biomarkers would further improve the diagnostic accuracy for NPC. The predicted probability for NPC using the combination of two autoantibodies and VCA-IgA was calculated by: ln (*p*/(1 − *p*)) = 6.909 × (PRDX2) +2.757 × (PRDX3) + 3.107 × (VCA-IgA) − 2.414. As expected, ROC analysis illustrated that measurement of both autoantibody and VCA-IgA increased the diagnostic accuracy for NPC and early-stage NPC, compared with the test of the autoantibody or VCA-IgA alone (Fig. 4; Table 3).

### The correlation of autoantibody assay positivity with Clinicpathological parameters

We evaluated the correlation of positive rates of the autoantibody assay with clinical variables in NPC patients. We did not find a correlation of assay positivity with any of the clinicpathological parameters of NPC patients (Table 4).

**Table 4:** Association of positive rates of PRDX2 autoantibody and PRDX3 autoantibody with NPC patients’ clinicopathologic characteristics

	n	PRDX2 autoantibody			PRDX3 autoantibody			Combination		
		Positive (%)	*χ* ^2^	*P*	Positive (%)	*χ* ^2^	*P*	Positive (%)	*χ* ^2^	*P*
Gender										
Male	100	26 (26.0%)	1.564	0.211	25 (25.0%)	0.009	0.925	34 (34.0%)	1.138	0.286
Female	29	11 (37.9%)			7 (24.1%)			13 (44.8%)		
Age										
≤50	61	19 (31.1%)	0.344	0.558	16 (26.2%)	0.126	0.723	21 (34.4%)	0.201	0.654
>50	68	18 (26.5%)			16 (23.5%)			26 (38.2%)		
T stage										
T1 + T2	65	16 (24.6%)	1.059	0.303	19 (29.2%)	1.375	0.241	27 (41.5%)	1.474	0.225
T3 + T4	64	21 (32.8%)			13 (20.3%)			20 (31.3%)		
N stage										
N0 + N1	67	20 (29.9%)	0.093	0.760	15 (22.4%)	0.437	0.509	25 (37.3%)	0.047	0.829
N2 + N3	62	17 (27.4%)			17 (27.4%)			22 (35.5%)		
M stage										
M0	122	34 (27.9%)		0.408*	30 (24.6%)		1.000*	44 (36.1%)		0.705*
M1	7	3 (42.9%)			2 (28.6%)			3 (42.9%)		
Overall stage										
I + II (early stage)	40	11 (27.5%)	0.040	0.842	10 (25.0%)	0.001	0.973	16 (40.0%)	0.318	0.573
III + IV (advanced stage)	89	26 (29.2%)			22 (24.7%)			31 (34.8%)		

*NPC* nasopharyngeal carcinoma

Statistical significance was determined by means of Chi-squared test or Fisher’s exact test (*)

## Discussion

In this study, we found novel TAAs in NPC cell lines (CNE2) and related autoantibodies in serum of patients with NPC using SERPA. We then identified autoantibodies against PRDX2 and PRDX3 and verified their diagnostic values in 129 patients with NPC and 100 normal controls. Importantly, combined testing of the two autoantibody biomarkers and VCA-IgA in serum could provide improved result for diagnosing NPC.

The present study identified 14 tumor autoantibodies that might serve as potential biomarkers for NPC. To our knowledge, we first showed the four proteins, Serine-threonine kinase receptor-associated protein, Gamma-glutamylcyclotransferase, Serpin B5 and PRDX3, could induce autoantibodies among cancer patients. Using a validation cohort, we demonstrated that two of these biomarkers (autoantibodies against PRDX2 and PRDX3) represent novel autoantibody targets for discriminating NPC from normal controls. Interestingly, both of PRDX2 and PRDX3 identified are members of the *Peroxiredoxin* (PRDX) gene family [23]. Peroxiredoxins are a family of antioxidant enzymes, which are ubiquitously expressed and regulate levels of intracellular H_2_O_2_ by catalyzing reduction to water [24]. This highly conserved PRDX family participates in cellular antioxidant defense, and is also associated with cell signaling pathways involving cell proliferation, differentiation, apoptosis, and DNA damage [25, 26]. Several reports have mentioned PRDXs as tumor-associated antigen inducing autoantibody production in malignant tumor [13, 18, 27–29]. Ren et al. [27] demonstrated that positivity of autoantibody against PRDX1 was observed in sera from 9 of 68 (13.2%) patients with esophagus squamous cell carcinoma (ESCC), whereas no such activity was detected in 89 (0%) normal individuals. We previously showed that autoantibody against PRDX6 could serve as a potential serum biomarker for early detection of NPC and esophageal cancer [13, 18]. The present study was the first to show the presence of autoantibodies against PRDX2 and PRDX3 in sera from patients with NPC. We provided evidence that autoantibody against PRDX2 and PRDX3 could detected early-stage NPC (Table 3), showing their potential utility in early diagnosis of NPC.

The detection of cancer at early stage would contribute to the treatment and prognosis of cancer patients. There are evidences that the screening programs for the early detection of tumors, such as colorectal, prostate, breast, and lung cancer, can reduce mortality [30–32]. Although EBV DNA and antibodies against EBV antigens (e.g. VCA-IgA) are clinically used for NPC for many years, they may be of limited use as general screening test for NPC due to low specificity for distinguishing NPC from other EBV-related diseases in endemic regions and high false positive rate for primary screening [33–35]. A reliable biomarker-based assay as a supplement to EBV DNA/VCA-IgA for the early NPC diagnosis is highly desirable. In recent years, autoantibodies show promising value of clinical application in terms of the early detection of cancer [13–19, 36–38]. For example, *EarlyCDT*-Lung, as a potential complementary tool to computed tomography, was the first autoantibody-based diagnostic biomarker to be performed for the early detection of lung cancer in routine clinical practice [36–38]. For a screening tool to be useful, the sensitivity and specificity would be as high as possible. However, to qualify as a clinically useful marker/marker panel for initial detection of tumor disease, a molecular tumor marker/marker panel must have better diagnostic performance (i.e. sensitivity and specificity) than tumor markers currently used. We here measured the combination of autoantibodies against PRDX2 and PRDX3 and VCA-IgA in early-stage NPC and normal controls resulting in 50.0% sensitivity with a robust specificity of 95.0% (Table 3). The combined assay comprising VCA-IgA and autoantibodies against PRDX2 and PRDX3 demonstrated a better diagnostic ability than each one tested alone. Thus, the addition of autoantibodies, one kind of simple and cost-effective biomarkers, may improve the diagnostic efficacy for NPC. The high specificity suggests that this combined detection might also be used in assay positive patients to monitor therapy response or alternatively as a tool to detect recurrence. On the other hand, the sensitivity of this test would be not high enough to be used for screening purposes in general or high risk populations. Thus, we need to identify new autoantibody markers [e.g. other potential autoantibody markers identified in the present study (shown in Table 2)] that could enhance the sensitivity of our present combined assay.

It is unclear what is the basis for the humoral responses to PRDX2 and PRDX3 antigens in NPC. Generally, cancer-associated autoantibodies target important protein molecules involved in carcinogenesis, which are deemed to be overexpressed, mutated, misfolded, or aberrantly modified in tumor cells [39, 40]. Overexpression of PRDX2 and PRDX3 has been reported in many kinds of cancer though it’s low or even undetectable in normal tissues [41, 42]. However, whether the autoimmune responses to PRDX2 and PRDX3 in NPC originate from its overexpression or other ways remains to be investigated.

## Conclusions

To the best of our knowledge, we are the first to identify autoantibodies against PRDX2 and PRDX3 by a proteomic approach in sera from NPC patients. Our results reveal that autoantibodies against PRDX2 and PRDX3 might serve as a potential supplement to VCA-IgA in NPC diagnosis. The combined detection of VCA-IgA and autoantibodies against PRDX2 and PRDX3 had a better diagnostic sensitivity than VCA-IgA tested alone (Table 3; Fig. 4) and a robust specificity, indicating that this test might make a contribution to the diagnosis and screening of NPC patients. However, due to the small size of patients with early disease in the present study, we need to further address the early diagnostic value of this test in a large cohort study. Another limitation is that we just included 7 NPC patients with advanced disease in the discovery work. We would conduct comparative analysis of NPC Stage I/II versus Stage III/IV versus control to discover early autoantibody biomarkers in the future work. What’s more, our observations also suggest that we should conduct further study to validate the diagnostic ability of other autoantibodies identified in this study. We hope to establish and validate an optimized autoantibody panel with VCA-IgA in larger, blinded patient cohorts obtained from multiple institutions, which could have clinical and economical implications for NPC screening.

## Abbreviations

AUC: area under the ROC curve
CI: confidence interval
ELISA: enzyme-linked immunosorbent assay
EBV: Epstein–Barr virus
HRP: horseradish peroxidase
NC: normal controls
NPC: nasopharyngeal carcinoma
NLR: negative likelihood ratio
NPV: negative predictive value
PVDF: polyvinylidene fluoride
PLR: positive likelihood ratio
PPV: positive predictive value
ROC: receiver operating characteristic
SERPA: serological proteome analysis
TAA: tumor-associated antigen
2-DE: two-dimensional gel electrophoresis
VCA-IgA: viral capsid antigen immunoglobulin A
